# Patients’ Coping Behaviors to Unavailability of Essential Medicines in Primary Care in Developed Urban China

**DOI:** 10.15171/ijhpm.2020.09

**Published:** 2020-01-27

**Authors:** Dan Wu, Tai Pong Lam, Kwok Fai Lam, Kai Sing Sun, Xu Dong Zhou

**Affiliations:** ^1^Department of Family Medicine and Primary Care, The University of Hong Kong, Hong Kong, China.; ^2^Department of Clinical Research, Faculty of Infectious and Tropical Diseases, London School of Hygiene and Tropical Medicine, London, UK.; ^3^Department of Statistics and Actuarial Science, Faculty of Science, The University of Hong Kong, Hong Kong, China.; ^4^Duke-NUS Medical School, Singapore, Singapore.; ^5^Institute of Social Medicine, School of Public Health, Zhejiang University, Zhejiang, China.

**Keywords:** Essential Medicines Policy, Primary Care, Service Utilization, China

## Abstract

**Background:** China rolled out the national essential medicines policy (NEMP) in primary care in 2009 and led to some unintended consequences including unavailability of essential medicines. This study examined patients’ coping behaviors to these unintended consequences of NEMP as well as the potential impact on primary care system development in Hangzhou, a developed city of China.

**Methods:** We conducted qualitative interviews and surveys with service users, primary care physicians (PCPs), and specialists in tertiary hospitals. Qualitative findings informed the design of the survey questionnaires. Main outcomes included patients’ coping behaviors after the NEMP implementation, as well as providers’ perceptions of NEMP’s impact on primary care development. Thematic analysis of the qualitative data and descriptive analysis of the survey data were conducted.

**Results:** Unintended effects of NEMP included frequent unavailability of certain essential drugs, leading to patient flow from primary care to hospital outpatient clinics for drug refills, difficulties in the provision of continuing care in primary care, as well as compromised patient trust in PCPs. In total, 1248 service users completed the questionnaires. A total of 132 (10.6%) were aged 60 years or above. Among 153 (57.7%) of the 265 who had some chronic condition(s) and needed long-term medication treatment, 60.1% went to hospitals for refills. Four-hundred sixty PCPs and 651 specialists were recruited. Among 404 PCPs who were aware of the NEMP policy implementation in their facility, 169 (41.8%) reported that there was often a shortage of drugs at their facilities and 44 (10.9%) reported always. Moreover, 68.6% of these PCPs thought that the NEMP could not meet their patients’ needs. Further, 44.2% (220/498) of specialists who were aware of the NEMP policy in primary care reported that they often heard patients complaining about the policy. In total, 53.1% of PCPs and 42.4% of specialists disagreed that NEMP helped direct patient flow to community-based care.

**Conclusion:** NEMP’s unintended effects undermined patients’ utilization of primary care in a developed city in China and led to unnecessary hospital visits. Countermeasures are needed to mitigate the negative impacts of NEMP on the primary care system.

## Background

Since the marketization of health services in 1980s, drug sales have been an important source of revenue for health facilities in the public sector in China, accounting for more than 40% of a health facility’s income.^1,2^ As a result, over-prescription of profitable drugs and irrational drug use by medical professionals are widespread.^3^ To address the challenge of irrational drug prescriptions and ensure affordability of health services, one of the top 5 priorities of China’s health reforms was the rollout of a national essential medicines policy (NEMP) in primary care,^4^ which aims to ensure access to safe, effective, affordable medicines for all.^5^ In primary care facilities that have implemented NEMP, profit margins of drugs enlisted are not allowed (ie, zero-profit policy) and all drugs are covered by health insurance schemes. The reimbursement rates for essential drugs are significantly higher than those of non-essential drugs and primary care physicians (PCPs) are encouraged to use the listed essential medicines.

NEMP is now implemented in all government-run primary care facilities in China. In the 2012 national essential medicines list for primary care, 520 generic medicines were included based on disease epidemiology, clinical use, affordability, and availability of medicines, including 317 western medicines and 203 Chinese medicines.^6^ Provinces were allowed to have additional drugs on the list based on their own financial capabilities. Provincial level online bidding system and centralized procurement system for essential drugs has been established to reduce the inflated drug prices that were due to intermediate procedures. The central government sets the ceiling prices for essential drugs, and pharmaceutical companies then bid online. The winning companies are added to a list of providers of essential drugs on a provincial online platform. The online platform facilitates the central procurement and distribution of drugs between primary care facilities and pharmaceutical manufacturers.

Effects of the NEMP have been widely evaluated. The vast majority of publications focused on the impact of NEMP on medical expenditures,^7-14^ rational use of medications,^8,11,14-16^ availability and affordability of essential medicines,^9,14,17-19^ by examining prescription data from various tiers of health facilities in the public sector as well as surveying availability of essential medicines in pharmacies in the private sector.^17^ Poor availability of essential medicines has been frequently reported as an unintended effect of NEMP implementation.^19^ However, voice of PCPs and patients have been neglected. In the past 5 years, only one 2016 study examined PCP and patient satisfaction with NEMP.^20^ Further, a limited number of Chinese publications reported increased service use both at primary care level and hospitals after the NEMP implementation.^21,22^ But none examined the patient flow between different tiers of health facilities and what factors might be driving the patient flow. How primary care patients cope with the unavailability of essential medicines and how the primary care system might be affected merit more research.

Primary care is the health system’s bedrock. The benefits of good primary care to health systems include improved population health outcomes, increased health system efficiency, and reduced health inequality between different income groups.^23,24^ China has a 3-tier healthcare system. Primary care facilities are expected to deal with common, simple conditions, while secondary and tertiary hospitals provide specialist referral care. Nevertheless, it is not mandatory for patients to go through primary care facilities, and patients can bypass primary care and see specialists at secondary and tertiary hospitals. Given most quality resources, including physicians with more formal medical education, are concentrated in hospitals due to the competition between different tiers of health facilities,^25^ many patients prefer hospital outpatient services which are widely believed to have better quality.^26,27^ China’s primary care system is facing challenges in attracting and retaining patients. Underutilization of primary care and abuse of hospital resources are substantial challenges for China’s health system.^25,28^ Therefore, to improve the health system efficiency, another top priority of the health reforms is to strengthen the primary care system with a goal of establishing an effective gatekeeping mechanism.^4^ Health reform policies complexly interact each other and their impact on one another should be evaluated.

This study aimed to examine, from service users and medical professionals perspectives, patients’ coping behaviors to the unavailability of essential medicines at community level, and the effects of NEMP on primary care system in the Chinese settings. We adopted a mixed methods approach, using both qualitative interviews and subsequent surveys with the service users, PCPs, and specialists. The study findings have implications for China’s future NEMP and primary care reforms or other low- and middle-income countries with similar contexts.

## Methods

### Study Design

This research project consisted of 2 phases—Phase 1 (a qualitative study) and Phase 2 (a quantitative study). The study design was published elsewhere.^26,29^ In Phase 1, semi-structured individual interviews and focus group discussions were conducted to elicit detailed accounts from both service users and service providers. Topic guides were developed for interviews based on literature review. In Phase 2, questionnaire surveys with service users and physicians were carried out to triangulate findings that emerged from the qualitative study. The Phase 1 qualitative study informed the design of the questionnaire.

### Study Population

The developed capital city of Zhejiang province, Hangzhou, had a population of 6.95 million in its eight districts in 2014-2015 when data were collected. In 2014, there were 92 comprehensive hospitals, 120 community health centers, and 905 satellite stations. The target populations of this study were the general public residing in the city, and medical professionals working at comprehensive hospitals and community health facilities (including centers and their affiliated satellite stations) in Hangzhou.

### Sampling and Data Collection

#### Qualitative Study

The qualitative study (Phase 1) was conducted from September 2014 to April 2015. Purposive sampling was adopted to recruit participants. A local researcher, who had strong connections with local health authorities, was identified and interviewees were approached based on professional networks.

The sampling framework for the service users included their age, education levels, income levels, and whether they were diagnosed as having chronic conditions. Group interviews were mainly arranged based on age groups. Sampling strategy for medical professionals encompassed the following characteristics: clinical settings, facility location, and professional ranking. Physicians were grouped based on their professional ranking to avoid hierarchy in the same group. Recruitment ceased when data saturation was reached.

We asked patients about their experiences of purchasing medications at community health facilities and doctors about their views of NEMP’s impact on patients. The interviewers were unknown to most participants and the researchers maintained their neutrality during the discussions. Most interviews lasted around 60 minutes and took place in private rooms. All interviewees were informed of the purpose of the study, and anonymity and confidentiality were assured. Consent was obtained before the interviews. An incentive of 50 Chinese Yuan (US$7.22) was offered to each participant as a token of gratitude.

#### Quantitative Study

The quantitative study (Phase 2) was conducted from July to September 2015. Multi-stage stratified random sampling was used to recruit service users.^26^ We asked them about their demographic backgrounds, whether they had any chronic conditions that required long-term medication treatment, availability of medications at primary care facilities, and visits to hospital facilities.

We used multi-stage stratified sampling to sample PCPs from community health facilities.^29^ A total of 12 community health centers were included and all PCPs present on surveying days were invited. An invitation letter with the link to the online survey sealed within an envelope was distributed to PCPs. Three comprehensive tertiary hospitals in central Hangzhou were conveniently selected for recruiting specialists. Specialists present on surveying days were invited the same way as PCPs. The online questionnaire could be filled in with an electronic device by scanning a QR code or inputting the link provided in the letter in a browser. We asked physicians about their demographic information, views towards availability of essential drugs, and NEMP’s impact on patient flow.

Respondents were told that the survey was anonymous and voluntary. No personal particulars were recorded, and confidentiality was strictly protected. A 5-Yuan (US$0.72) incentive was provided to each respondent.

### Data Analysis

Interviews were audio recorded and transcribed. One of the authors checked the accuracy of the transcripts. A thematic framework analysis was conducted using NVivo 12. Codebooks were developed based on the first 2 interviews and then were used to guide the rest of the analysis. Two investigators coded the data independently. The coding consistency between the 2 sets was checked. Discrepancies were then discussed and resolved by the 2 investigators. Quotes were translated from Chinese into English. The key themes that emerged were then incorporated into the questionnaire for the survey. The survey data were analyzed using SPSS Version 25. Descriptive analysis was carried out to describe respondents’ views towards NEMP policy and its impact on service utilization.

## Results

### Qualitative Findings From Service Users

We conducted 13 semi-structured individual interviews and 6 focus group discussions (6 to 8 participants per group). Among the participants, 44.4% were men. The mean age of all participants was 52.6 years (standard deviation [SD] = 17.0). The most frequent complaint revealed by service users was the unavailability of medications at community health facilities, including some commonly used medications and imported ones (Supplementary file 1, quotations 1-3). Service users said that this caused inconvenience, and they had to go to a hospital just to get medications. The perceived unavailability of certain essential medicines at primary care facilities had a negative impact on service users’ intention to use community-based care (Supplementary file 1, quotation 4).

### Quantitative Findings From Service Users

We recruited 1248 service users and their characteristics were reported elsewhere.^26^ Women accounted for 49.4% of the total respondents (Table 1). Most respondents (769, 61.6%) were aged below 40 years and 132 (10.6%) were aged 60 years or above. Totally, 1037 (83.1%) reported being covered by some health insurance. Among all, 265 (21.2%) reported some chronic condition(s) that had been diagnosed earlier, and 153 (57.7%) of them needed long-term medication treatment. Among these 153 respondents, 60.1% went to hospitals for drug refills.

**Table 1:** Characteristics of Service User Respondents (N = 1248)

**Variables**	**No. (%)**
Gender	
Male	578 (46.3)
Female	616 (49.4)
Missing	54 (4.3)
Age	
18-39	769 (61.6)
40-59	298 (23.9)
60 or above	132 (10.6)
Missing	49 (3.9)
Education	
High school or less	694 (55.6)
Post-secondary education or higher	504 (40.4)
Missing	50 (4.0)
Employment status	
Employed	813 (65.1)
Retired	193 (15.5)
Unemployed	185 (14.8)
Missing	57 (4.6)
Monthly income (US$)	
<578	304 (24.4)
578-1444	462 (37.0)
>1444	393 (31.5)
Missing	89 (7.1)
Insurance status	
Not insured	140 (11.2)
Insured	1037 (83.1)
Missing	71 (5.7)
Diagnosed chronic conditions	265 (21.2)
Needed long-term medication (n = 265)	153 (57.7)
Availability of medications at the community (n = 153)	72 (47.1)
Went to hospitals to refill medications (n = 153)	92 (60.1)

### Qualitative Findings From Physicians

Five focus group discussions with PCPs at community health facilities (4 to 8 PCPs in each group) and 2 groups with specialists from 3 tertiary hospitals (6 and 8 specialists respectively) were conducted. Semi-structured individual interviews were conducted with 13 PCPs and 8 specialists. Sixty-one percent of the participants were men. The mean age of all physicians was 34.3 years (SD = 5.8).

The interviews with PCPs also revealed the unavailability of certain drugs for common diseases. They perceived clinically unmet patients’ needs due to the limited number of drugs available at community health facilities, especially among patients with multiple morbidities (Supplementary file 2, quotation 1). Patients then had to go to a hospital for medications they needed. The specialists’ experiences and perceptions triangulated the service users’ and the PCPs’ views. A specialist working in one of the tertiary hospitals complained that she had to write prescriptions for patients to get their refills (Supplementary file 2, quotation 2).

The PCPs articulated that it was the NEMP policy as well as its accompanying regulations that led to the above issues. Such unexpected effects were conflicting with the goal of promoting primary care’s gatekeeper role (Supplementary file 2, quotations 3 and 4). Reasons for why the implementation of NEMP led to such coping behaviors were discussed. One major factor was the exclusion of imported medications to ensure the affordability of essential drugs. Imported medications were regarded to have better quality. However, primary care facilities were unable to provide refills of imported drugs that were prescribed by specialists (Supplementary file 2, quotations 5 and 6). Such an issue also compromised the patient’s trust in primary care providers’ capability to cure patients, as was illustrated by a PCP (Supplementary file 2, quotation 7).

### Quantitative Findings From Physicians

A total of 1111 physicians completed the questionnaire, including 460 PCPs from community health facilities and 651 hospital specialists (Table 2). Characteristics of the physicians were reported elsewhere.^29^ The mean age was 34.5 years (SD = 7.5). Women accounted for over 60% of the respondents in both groups. Overall, they had an average of 10.7 years of practice (SD = 8.3). Specialists, in general, had higher educational attainments than PCPs (*P* <.001, Pearson chi-square test).

**Table 2:** Demographic Characteristics of the Physician Respondents

	**No. (%)** **n = 1111**		**PCPs (%)** **n = 460**		**Specialists (%)** **n = 651**		***P*** ** Value**
Age (mean, SD)	34.5	(7.5)	36.4	(6.8)	33.2	(7.7)	<.001
Years of practice (mean, SD)	10.7	(8.3)	13.5	(8.1)	8.7	(7.9)	<.001
Gender							0.09
Male	392	(35.3)	143	(31.1)	249	(38.2)	
Female	691	(62.2)	289	(62.8)	402	(61.8)	
Missing	28	(2.5)	28	(6.1)	0	(0)	
Education							<.001
Post-secondary or less	158	(14.2)	105	(22.8)	53	(8.1)	
University or higher	942	(84.8)	344	(74.8)	598	(91.9)	
Missing	11	(1.0)	11	(2.4)	0	(0)	

Abbreviations: PCPs, primary care physicians; SD, standard deviation.

#### PCPs’ Views

Table 3 shows PCPs’ views towards the NEMP policy. Over 88% of PCPs were aware that the NEMP policy had been implemented in their facility, with most of them reporting the implementation being over 2 years before the survey. Among the 404 PCPs who were aware of the policy, up to 278 (68.8%) thought that the NEMP could not meet their patients’ needs of medicine. Out of 404, 169 (41.8%) reported that there was often a shortage of drugs at their facilities and 44 (10.9%) reported always. Only a third (135, 33.5%) considered NEMP as effective in directing patients to community-based care, while 214 (53.1%) disagreed.

**Table 3:** PCPs’ Views Towards the Effects of the Essential Drug List on Patients’ Help-Seeking

	**No.**	**%**
For how long has essential drug list policy been implemented at your workplace? (n = 460)		
Not implemented yet	7	1.5
Within 1 year	20	4.4
1-2 years	39	8.6
2-4 years	176	38.9
>4 years	163	36.1
Don't know	47	10.4
From the medical perspective, to what degree can the essential drugs satisfactorily meet patients' needs? (n = 404)		
Unsatisfactory at all	72	17.8
Unsatisfactory	205	50.8
Uncertain	12	3.0
Satisfactory	108	26.7
Very satisfactory	5	1.2
Don't know	2	0.5
How often is there a shortage of drugs at your community facility? (n = 404)		
Never	17	4.2
Occasionally	167	41.3
Often	169	41.8
Always	44	10.9
Don't know	7	1.7
How often do you hear about patients' complaints against the essential drug list policy? (n = 404)
Never	5	1.2
Occasionally	100	24.8
Often	220	54.5
Always	79	19.6
Does the current essential drug list policy help direct patients to community-based care? (n = 404)		
Yes	135	33.5
No	214	53.1
Don't know	54	13.4

Abbreviation: PCPs, primary care physicians.

#### Specialists’ Views

Specialists’ views of the effects of NEMP are shown in Table 4. Slightly over 3-quarters (76.5%) were aware of the implementation of NEMP at community health facilities near their hospital. Among them, more than half (53.5%) thought that NEMP failed to meet patients’ needs in primary care. One-third perceived that there was often/always a shortage of drugs. A total of 220 (44.2%) reported that they often heard patients complaining about the NEMP policy. Overall, slightly over a third (181, 36.3%) agreed that NEMP helped divert patients to primary care, while 211 (42.4%) disagreed.

**Table 4:** Specialists’ Views Towards the Effects of the Essential Drug List on Patients’ Help-Seeking

	No.	%
Has essential drug list policy been implemented at community health facilities in the area where your hospital is located? (n = 651)		
Yes	498	76.5
No	37	5.7
Don't know	116	17.8
From the medical perspective, to what degree can the essential drugs satisfactorily meet patients' needs in primary care? (n = 498)		
Unsatisfactory at all	76	15.3
Unsatisfactory	190	38.2
Uncertain	59	11.8
Satisfactory	149	29.9
Very satisfactory	6	1.2
Don't know	18	3.6
How often is there a shortage of drugs at community health facilities around your workplace? (n = 498)		
Never	19	3.8
Occasionally	216	43.4
Often	144	28.9
Always	22	4.4
Don't know	97	19.5
How often do you hear about patients' complaints against the essential drug list policy? (n = 498)		
Never	20	4.0
Occasionally	234	47.0
Often	190	38.2
Always	30	6.0
Does the current essential drug list policy help direct patients to community-based care? (n = 498)		
Yes	181	36.3
No	211	42.4
Don't know	106	21.3

## Discussion

NEMP is an important strategy to ensure accessibility and affordability of safe and effective drugs in China’s primary care system. Evaluation of the impact of NEMP on the development of primary care system is however lacking. This study extends the literature by identifying patients’ coping behavior to unavailability of essential medicines by visiting hospital outpatient services, leading to unnecessary patient flow from primary care to hospital-based care. We found other negative impact of NEMP on the primary care system including difficulty in providing continuing care and compromised patient trust in PCPs.

Our study found that the unavailability of essential medicines affected patient access to medications in primary care and caused patient complaints. This is consistent with an earlier study which found patient dissatisfaction with the quantity of essential medicines in another province, Ningxia.^20^ Exclusion of medicines with higher effectiveness but more expensive, reduced quality and quantity of essential medicines to avoid financial loss by pharmaceutical manufacturers were frequently reported contributing factors.^19^ Our study also found that people from developed urban areas had expectations of medicines of better effectiveness, such as imported ones. In addition, many Chinese patients prefer hospital-based outpatient services for an initial diagnosis and management plan.^26^ However, it is not mandatory for hospital specialists to prescribe essential medicines,^18^ and they often prescribe medications which are believed to have better quality but less likely to be available in primary care, causing challenges for PCPs to refill the same medicines. PCPs may also find it challenging to change the medication regime because of patient distrust.^29^ As a result, both patients’ help-seeking autonomy and PCPs’ prescribing autonomy are compromised to some extent, contributing to discontent among both groups.

As a result, patients, especially those with chronic conditions who need long-term medical treatment, adapted their healthcare seeking pattern by visiting hospital outpatient clinics for drug refills. Over 60% of service user respondents who needed long-term medications reported visiting hospitals for drug refills. PCPs and hospital specialists conveyed the same message. Being an alternative for patients to refill, pharmacies are facing the similar challenge of unavailability of some essential medicines.^19^ They may also be less appealing owing to lower patient trust and patients’ preference for a follow-up medical consultation at the same time. This unavailability consequently drove many patients away from community-based primary care facilities to hospitals, potentially leading to wastage of hospital resources. Such a trend of patient flow runs counter to other goals of the national health reforms such as promoting primary care and reducing wastage of hospital resources.^4^

Lastly, other unintended impact of NEMP on China’s primary care system were compromised patient trust in PCPs and difficulty in provision of continuing care by PCPs. A 2013 study reported that primary care doctors were scolded by patients for not being able to prescribe medicines they needed.^30^ Our participants further revealed that unavailability of essential drugs negatively affected service users’ trust in PCPs’ clinical competence. Patient trust is essential to the physician-patient relationship and for retaining patients in primary care. Due to less formal medical education among PCPs and concentration of qualified physicians in hospitals, China’s primary care system is facing challenges in gaining patient confidence and attracting patients to community-based facilities.^31^ Such an unanticipated impact on patient trust may further compound the PCP-patient relationship issue. Providing effective continuing care to patients is a key function of a healthy primary care system. Our study demonstrated that many patients intended to seek follow-up care at community health facilities but were deterred away to hospitals. The continuity of care at primary level deteriorated to some extent because of the unavailability of essential medications.

To mitigate the negative impact on primary care system and retain patients at the community level, actions are needed. The National Health Commission of the People’s Republic of China increased the number of essential drugs from 520 to 685 in the most updated version of NEMP in 2018.^32,33^ In addition to this, various provincial health bureaus are also gradually relaxing the policy in primary care. For example, over 10 provinces and regions including Zhejiang, Chongqing, Gansu, and Shandong, have partially relaxed the NEMP and allowed 30%-50% of their drugs to be non-essential in primary care facilities.^34^ Public awareness campaign to improve service users’ understanding of the NEMP may be helpful to mitigate the risk of deteriorated distrust in PCPs. Patient preferred sources for first-contact care should also be considered in future policy changes given the discrepancies in NEMP implementation and disease profile between primary care facilities and hospitals. Taking economic backgrounds of local populations into account, allowing more non-essential drugs in primary care facilities, and further strengthening the role of pharmacies for drug refills in the private sector may be solutions to better meet patients’ needs in urban cities. Actions are also necessary to maintain good quality of essential medicines produced by pharmaceutical companies and secure supply to both primary care facilities and pharmacies.

Our study has some limitations. First, we conducted the study in a developed urban area in China. The study results may not be representative of other poorer regions in the country. However, our findings may reflect the situation in Chinese cities with relatively better-off economic conditions. Second, self-administered questionnaire surveys might introduce recall bias regarding past experiences. Most items solicited respondents’ views towards their recent experiences and recall bias is likely to be inconsequential. Additionally, we recruited a slightly lower proportion of people aged 60 or above and the findings should be interpreted with caution. Third, we were not able to obtain more concrete service utilization data owing to lack of access to government and clinical data. Self-reported responses at a certain point in time have limited power to reflect the changing pattern of dynamic service utilization. However, this study provides a snapshot of the effects of NEMP on service use and service delivery in primary care settings from both service users’ and providers’ perspectives and can serve as the starting point of monitoring the impact on primary care system. Lastly, random sampling of hospital specialists was challenging. There was a selection bias in sampling specialists and the representativeness of specialist views may be compromised.

## Conclusion

NEMP policy is a promising strategy to improve accessibility and affordability of medicines in low- and middle-income countries. Our study investigated NEMP’s impact on primary care system in China. We found that the unintended unavailability of essential medicines consequently deterred patients who needed long-term medical treatments from attending primary care, leading to unnecessary hospital outpatient visits for drug refills, as well as wastage of hospital resources. Discontent from both patients and service providers was evident. Such discontent might consequently compound the issue of building patient confidence in PCPs and bring more challenges in establishing effective gate-keeping mechanism and delivering continuing care at primary care level. Actions are needed to mitigate the negative impact of NEMP on the development of primary care system, and close monitoring of the impact of NEMP on patient service utilization is necessary.

## Acknowledgements

The work was supported by Committee on Research and Conference Grants of the University of Hong Kong, Hong Kong, China [Grant Number 201409176026]. The funder played no role in the design of the study, data collection, data management, data analysis and interpretation, preparation, review, and approval of the manuscript.

## Ethical issues

Ethical approvals were obtained from the local Institutional Review Board of The University of Hong Kong/Hospital Authority Hong Kong West Cluster (UW14-567) and Zhejiang University (ZGL201410H).

## Competing interests

Authors declare that they have no competing interests.

## Authors’ contributions

DW and TPL conceived and designed the study. XDZ and DW collected data. DW, SKS, and KFL analysed data. DW, SKS, and TPL drafted the paper and other authors provided constructive suggestions and edited the paper. All authors have seen and approved the final version of the paper.

## Authors’ affiliations

^1^Department of Family Medicine and Primary Care, The University of Hong Kong, Hong Kong, China. ^2^Department of Clinical Research, Faculty of Infectious and Tropical Diseases, London School of Hygiene and Tropical Medicine, London, UK. ^3^Department of Statistics and Actuarial Science, Faculty of Science, The University of Hong Kong, Hong Kong, China. ^4^Duke-NUS Medical School, Singapore, Singapore. ^5^Institute of Social Medicine, School of Public Health, Zhejiang University, Zhejiang, China.

## Supplementary files

Supplementary file 1. Impact of NEMP on Availability of Medications at Community Health Facilities From Service Users’ Perspective.

Supplementary file 2. Physicians’ Views Towards NEMP’s Impact.

## Key messages

**Implications for policy makers**

- The implementation of national essential medicines policy (NEMP) in primary care has unexpectedly led to patient flow from primary care to hospital-based services for drug refills, causing health resources wastage.
- The shortage of essential drugs created difficulties in primary care physicians’ (PCPs’) provision of continuous care and prescription autonomy, which further compromised patient trust in PCPs.
- In the context of intensive primary care reforms in China, evaluation of the impact of NEMP on patients’ utilization of primary care services merits more attention from policy-makers.

**Implications for public**

This study may help informing the public about the status quo of the National Essential Medicines Policy (NEMP) and mitigate to some extent the public distrust in primary care physicians (PCPs) due to misinterpretation of the unavailability of essential drugs in primary care. The study findings also reflected the public’s views towards the NEMP, which are often neglected by policy-makers. Having the public’s voice heard by a wider community, potentially including health reformers, provides an opportunity to incorporate their feedback into future NEMP reforms.
