# Supplementary material for: Patients’ Coping Behaviors to Unavailability of Essential Medicines in Primary Care in Developed Urban China
Source: Int J Health Policy Manag. 2020 Jan 27;10(1):14–21. doi: 10.15171/ijhpm.2020.09 (PMC7947706; doi:10.15171/ijhpm.2020.09)
Supplement: Supplementary file 1 — Impact of NEMP on Availability of Medications at Community Health Facilities From Service Users’ Perspective. [file ijhpm-10-14-Supp1.pdf]

Supplementary file 1. Impact of NEMP on Availability of Medications at Community Health Facilities From Service Users' Perspective

**Themes**	**Sub-themes**	**Quotations**
Impact on medications in primary care	Unavailability	1. The number of medications available is too small and there are no good drugs. I came here for 3 medications and all 3 were unavailable. The cheaper the medication, the less likely it is available. (Group1, patients, P3)<br>2. I have osteoporosis and have to take vitamin D. Xianlinggubao capsules (an industrially processed Chinese medicine), calcium tablet and pu li de (a Chinese commercial brand of glucosamine hydrochloride). Community [facilities] just don't have these. (Group1, patients, P2)<br>3. Here they don't have imported medications for diabetes. I am taking 2 capsules per day. But [I need] one capsule only if it is the imported drug. (Group1, patients, P5)
	Impact on primary care use	4. If I come today, and the medications I need are not available, after a couple of times, I won't come here again. (Group1, patients, P3)

Abbreviation: NEMP, national essential medicines policy.
