# Supplementary material for: Patients’ Coping Behaviors to Unavailability of Essential Medicines in Primary Care in Developed Urban China
Source: Int J Health Policy Manag. 2020 Jan 27;10(1):14–21. doi: 10.15171/ijhpm.2020.09 (PMC7947706; doi:10.15171/ijhpm.2020.09)
Supplement: Supplementary file 2 — Physicians’ Views Towards NEMP’s Impact. [file ijhpm-10-14-Supp2.pdf]

Supplementary file 2. Physicians' Views Towards NEMP's Impact

Themes	Sub-themes	Quotations
Impact on patients' use of healthcare	Primary care	1. Elderly people may have many diseases, including hypertension, diabetes, and coronary heart disease. They may indeed need many medications. But primary care facilities have only 300 generic drugs and these 300 kinds of drugs are the most common, of worst quality, and the cheapest. They cannot meet patients' demands at all. We might have a couple of drugs that the patients need but they must go to a hospital for the other 5 or 6 drugs. (Individual, PCP, P5)
	Hospital-based services	2. Take a hypertensive patient for example. He needs to take medications every day. When he finishes his medications, he can go to community facilities (for refills) and does not need to come to a hospital. But community facilities have fewer medications. It's called, ah, something like a … list [the physician was trying to recall the name]. Many patients come to our hospital to refill medications. We have a lot of patients already, but I also have to write prescriptions for patients to get refills. (Individual, specialist, P1)
Impact on the healthcare system	Primary care's gatekeeper role	3. Interviewer: Is this related to the national essential drug policy?<br>P11: Of course, it is.<br>Interviewer: What kinds of drugs needed by patients are not available here?<br>P11: Like some insulin pens. We only have Novolin and don't have others, like those long-acting insulins. We have NovoRapid, but, before we prescribe it, patients must be diagnosed by an endocrinologist and the drug must be prescribed by the endocrinologist first. Then we are allowed to prescribe it. (Individual, PCP, P11)<br>4. There is nothing we can do about the national essential drug list policy. It strictly controls the list of drugs at community facilities. Now [the country] encourages the public to go to the community facilities for first-contact care. But we cannot match [with patients' needs]. This is a huge conflict. (Group 4, PCPs, P6)

Reasons for unavailability of essential drugs	Imported drugs	5. Regarding the quality of drugs, domestic and imported ones are different. Most big hospitals use imported drugs more often, but in community facilities, basically domestic ones [only]. So, medicines prescribed in big hospitals cannot be refilled at community facilities. And it is challenging [for PCPs] to change treatment medications. (Group1, specialists, P6)<br>6. For example, medications for asthma are not available in community facilities. Their drugs are basic and of poor quality, or those which should be discontinued. What can patients do then? Community facilities don't even have inhaler drugs. (Individual, specialist, P5)
Impact on patient-primary care provider relationship	Patient trust in primary care	7. In recent years, there has been a strict list of drugs, and many good drugs are not available at community facilities. The patients think that our community facilities do not even have the drugs, so we are not able to cure them. (Individual, PCP, P6)

Abbreviations: NEMP, national essential medicines policy; PCP, primary care physician.
